# Gram Negatives and Antimicrobial Resistance: Two Faces of the Same Coin

**DOI:** 10.3390/jcm11195574

**Published:** 2022-09-22

**Authors:** Nicola Petrosillo, Guido Granata

**Affiliations:** 1 Infection Prevention & Control/Infectious Disease Service, Fondazione Policlinico Universitario Campus Bio-Medico, 00127 Rome, Italy; 2 Systemic and Immune Depression-Associated Infection Unit, National Institute for Infectious Diseases “L. Spallanzani”, IRCCS, 00149 Rome, Italy

The evolution, emergence and spread of bacterial antimicrobial resistance (AMR) represent threatening healthcare concerns of worldwide proportions. Resistance to antimicrobials, both innate and acquired, is an older phenomenon than mankind and is part of the evolutionary adaptation of microorganisms to environmental modifications.

Indeed, researchers found in New Mexico a cave that has been isolated for over 4 million years, and inside this cave, they identified bacteria highly resistant to antibiotics; some strains were even resistant to 14 different currently commercially available antibiotics [1]. This therefore implies that antibiotic resistance has a long evolutionary past, in which non-pathogenic environmental organisms represent a reservoir of resistance genes that have the potential to be transferred to pathogens.

However, more recently, the adaptive evolutionary process has seen an acceleration, facilitated by pressure forces such as antimicrobial use and abuse in human, animal and environmental sectors and the spread of resistant bacteria and resistance determinants within and between these sectors and around the globe. These are currently the main drivers of antimicrobial resistance.

In this Editorial, we discuss a set of articles recently published on the Journal of Clinical Medicine, covering different areas of AMR, from multidrug-resistance in Gram negatives in urinary tract infections worldwide, to extensively drug-resistant (XDR) Pseudomonas aeruginosa bloodstream infections, to molecular methods for detecting antimicrobial resistances in Helicobacter pylori.

Although urinary infections are among the most common infections worldwide, data on antimicrobial resistance of the microorganisms causative of these infections are scant and in any case need to be updated. The article by Li X et al. [2] wishes to bridge this gap through the analysis of data on antimicrobial resistance (AMR) in urinary tract infections (UTIs) obtained from the Global Burden of Diseases, Injuries, and Risk Factors Study (GBD).

Led by the Institute for Health Metrics and Evaluation (IHME), the GBD study is the most comprehensive worldwide observational epidemiological study to date and provides an independent estimation of population, for each of 204 countries and territories and the globe, using a standardized, replicable approach.

In 2019, data from UTIs come from 7 super regions and 21 regions; these data refer to 14 pathogens, 13 antibiotic classes and 66 combinations of pathogen with antibiotics. Results from this study are amazing: 65 thousand and 0.26 million deaths are attributable to, and are associated with, bacterial AMR in UTIs.

Moreover, Southern and Tropical Latin America and Europe had the higher all-age deaths rate, whereas Sub-Saharan Africa had the lower rates; these findings appear paradoxical considering that in terms of the AMR burden for all infectious syndromes, the highest death rates occurred in the four sub-Saharan Africa areas. Probably, a low UTI burden in the Sub-Saharan Africa added to the scarcity of laboratory infrastructure, resulting in the lack of adequate quality of data, which can partly explain these findings. Of note, among the AMR-related causes of death globally, UTIs were fourth, behind lower respiratory infections, bloodstream infections and abdominal infections.

Finally, the analysis of GBD data evidenced that E. coli and K. pneumoniae accounted for around 50% of deaths due to AMR in UTI. Most resistant antibiotic classes included fluoroquinolones, carbapenems and third-generation cephalosporins, often in combination.

The knowledge of the regional distribution of UTI-causative microorganisms and their resistance in different areas and regions is crucial for informing location-specific programs aimed to control AMR and optimize the use of antibiotics in UTI. An analysis of region-specific organism–antibiotic interactions in terms of resistance will allow the design of tailored enhanced surveillance, antimicrobial stewardship programs and infection prevention and control strategies, as well as the proper allocation of resources.

If UTIs, especially when uncomplicated, are relatively easy to treat, bloodstream infections often represent a challenge due to the hematogenous spread of microorganisms in other body sites, causing infections such as endocarditis or osteomyelitis, or due to the immune response to infection causing sepsis or septic shock.

Among Gram negatives, Pseudomonas aeruginosa is particularly challenging due to its intrinsic antimicrobial resistance profile and its capacity to acquire resistance genes and to select chromosomal mutations.

Multidrug-resistant (MDR) or XDR Pseudomonas aeruginosa are increasingly being isolated in healthcare settings; the paucity or lack of therapeutic antimicrobial options pose patients with severe resistant P. aeruginosa infections at high risk for death.

While several data on mortality are available for carbapenem-resistant Pseudomonas aeruginosa, limited data are available for XDR strains. To address this issue, in a tertiary-care university hospital in Barcelona, Spain, Montero MM et al. retrospectively analyzed the clinical course of 122 bloodstream infections caused by XDR Pseudomonas aeruginosa and compared them with 260 non-XDR Pseudomonas aeruginosa bloodstream infections in the same hospital, in the same period [3]. In this center, ST175 high risk was the most common clone.

XDR Pseudomonas aeruginosa infections were significantly associated with nosocomial acquisition, more frequent in haematologic malignancy and catheter-related infection and received more often inappropriate empirical antimicrobial treatment. For XDR bloodstream infections, all-cause mortality at Day 30 was 33.6% (41/122), not significantly different from that in non-XDR bloodstream infections, i.e., 29.6% (77/260).

When the authors analyzed all variables associated with mortality in Pseudomonas aeruginosa bloodstream infections, both XDR and non-XDR, the independent risk factors for mortality included a high-risk source of infection, the presence of a septic shock and Pitt score; interestingly, appropriate definitive therapy was a significant protective factor for mortality.

This article, albeit with some limitations, showed that the prognosis of MDR Pseudomonas aeruginosa BSI depends on various factors beyond the resistance phenotype, including the severity of infection, having a high-risk source, the presence of different XDR phenotypes, virulence of the XDR high risk clone and theoretical fitness costs.

Despite all these limitations, it is noteworthy that one-third of patients with P. aeruginosa BSI will die and that the appropriateness of antibiotic therapy remains the major determinant of therapeutic success and survival.

In addition to E. coli, Klebsiella pneumoniae and Pseudomonas aeruginosa, another Gram-negative organism, is particularly threatening, i.e., Acinetobacter baumannii. Occurrence and spread of Acinetobacter baumannii, resistant to most of the available antimicrobial agents, including carbapenems, is of great concern since the treatment options become limited.

Acinetobacter baumannii is frequently associated with healthcare-acquired infections and causes a wide spectrum of infections, including pneumonia, bacteremia, meningitis, urinary tract infection and wound infection.

Mechanisms of resistance in A. baumannii are multiple and include (1) the presence of a wide array of beta-lactamases that hydrolyze and confer resistance to penicillins, cephalosporins and carbapenems; (2) the loss or the reduced expression of porins; the overexpression of efflux pumps for several drugs; (3) and, finally, alterations in penicillin-binding proteins. All these mechanisms render A. baumannii resistant to several antibiotics including carbapenems and susceptible only to colistin.

However, although rare, resistance to colistin has been described in A. baumannii. In A. baumannii, the main mechanism of colistin resistance is the modification of lipopolysaccharide (LPS) by mutations in PmrA/PmrB two-component system; moreover, there is also an intrinsic colistin tolerance mechanism associated with more than 30 genes.

Colistin has been for years the most used antimicrobial against MDR colistin-susceptible A. baumannii infections. In an article by Huang C. et al. [4], a meta-analysis of studies comparing the efficacy of colistin monotherapy versus the combination colistin plus meropenem in patients with MDR Acinetobacter baumannii infection was performed.

In this meta-analysis, three randomized controlled trials and seven retrospective studies were included. The combination of colistin and meropenem resulted not significantly superior to colistin alone; mortality was not different either. However, the combination group had a better microbiological response than the monotherapy (odds ratio 0.71, 95% confidence intervals 0.51–0.98). Of note, the A. baumannii strains in the studies included in the meta-analysis were MDR, XDR and carbapenem-resistant; moreover, no information on the pharmacokinetics of meropenem is available.

More recently, two promising drugs against A. baumannii have been introduced in clinical practice. Cefiderocol is a recent siderophore cephalosporin with a unique mechanism of action: it utilizes the siderophore–iron complex pathway to penetrate the outer membrane of Gram-negative organisms into the periplasmic space.

Cefiderocol is active against ESBL-producing, carbapenems-resistant Enterobacterales, including metallo beta-lactamases producers, and non-fermenting Gram-negative bacilli, including MDR A. baumannii strains.

Another promising drug active against A. baumannii is sulbactam, a well-known beta-lactamase inhibitor which also possesses antibacterial activity against A. baumannii, due to its selective binding to the PBP (1)–(3). Unfortunately, MDR A. baumannii strains emerged with a reduced expression of PBP2; these strains produce beta-lactamases that can degrade sulbactam. However, more recently, another beta-lactamase inhibitor, durlobactam, in combination with sulbactam showed high activity against beta-lactamases in Ambler Class A, C and D, thus representing a promising therapeutical option for MDR A. baumannii infections.

In the last decades, there has also been great interest in antimicrobial resistances in other Gram-negative organisms than the classical Enterobacterales or non-fermenting Gram negatives. Helicobacter pylori is a microaerophilic, Gram-negative bacterium that infects up to half of the population and is associated with various gastroduodenal diseases, including chronic active gastritis, peptic or duodenal ulcers, gastric adenocarcinoma and mucosa-associated tissue lymphoma. Its treatment includes antibiotics such as clarithromycin, metronidazole, amoxicillin, tetracycline, levofloxacin and rifabutin.

In the last years, likely due to antibiotic pressure, resistances are emerging in Helicobacter pylori, mainly for clarithromycin, metronidazole and quinolones. Beyond the classical bacterial culture and phenotypic drug susceptibility testing, there is a need for more rapid, cost-effective molecular assays enabling a reliable prediction of phenotypes of antibiotic resistance driving antimicrobial therapy against Helicobacter pylori.

Whole-genome sequencing (WGS) from cultured bacterial isolates represents currently an important tool for surveillance and antibiotic resistance control, and more recently, this technique showed that the occurrence of specific point mutations in genes of H. pylori enabled reliable prediction of drug resistance phenotypes, at least for macrolides, fluoroquinolones and rifamycin.

The articles of Lauener FN et al. [5] and of Tuan VP et al. [6] addressed all the unique features of WGS in predicting antibiotic resistance in Helicobacter pylori, both for clinical and for epidemiological purposes.

In conclusion, after the COVID-19 era, the next threatening pandemic is very likely represented by the global spread of MDR, XDR, pan-resistant Gram-negative strains causing severe infections with limited treatment options, with a considerable burden of morbidity and mortality. Surveillance, infection prevention and control at a One Health level (human, animal and environmental); knowledge of mechanisms of action of resistances; developing weapons against resistant strains; and strengthening awareness at a population level on the rational use of antimicrobials are the main elements for tackling this worldwide menace.
